# Methodological issues in economic evaluations of disease prevention and health promotion: an overview of systematic and scoping reviews

**DOI:** 10.1186/s12889-021-12174-w

**Published:** 2021-11-20

**Authors:** Yana Seleznova, Adrienne Alayli, Stephanie Stock, Dirk Müller

**Affiliations:** Institute for Health Economics and Clinical Epidemiology, Faculty of Medicine and University Hospital of Cologne, Gleueler Str. 176-178, 50935 Cologne, Germany

**Keywords:** Disease prevention, Health promotion, Economic evaluation, Methods, Systematic review, Health economics

## Abstract

**Background:**

We aimed to provide a comprehensive overview of methodological challenges in economic evaluations of disease prevention and health promotion (DPHP)-measures.

**Methods:**

We conducted an overview of reviews searching MEDLINE, EMBASE, NHS Economic Evaluation Database, Database of Promoting Health Effectiveness Reviews, Cochrane Database of Systematic Reviews (CDSR) and Database of Promoting Health Effectiveness Reviews (DOPHER) (from their inception to October 2021). We included both systematic and scoping reviews of economic evaluations in DPHP addressing following methodological aspects: (i) attribution of effects, (ii) outcomes, (iii) inter-sectoral (accruing to non-health sectors of society) costs and consequences and (iv) equity. Data were extracted according to the associated sub-criteria of the four methodological aspects including study design economic evaluation (e.g. model-based), type/scope of the outcomes (e.g. outcomes beyond health), perspective, cost categories related to non-health sectors of society, and consideration of equity (method of inclusion). Two reviewers independently screened all citations, full-text articles, and extracted data. A narrative synthesis without a meta-analysis or other statistical synthesis methods was conducted.

**Results:**

The reviewing process resulted in ten systematic and one scoping review summarizing 494 health economic evaluations. A lifelong time horizon was adopted in about 23% of DPHP evaluations, while 64% of trial-based evaluations had a time horizon up to 2 years. Preference-based outcomes (36%) and non-health outcomes (8%) were only applied in a minority of studies. Although the inclusion of inter-sectoral costs (i.e. costs accruing to non-health sectors of society) has increased in recent years, these were often neglected (between 6 and 23% depending on the cost category). Consideration to equity was barely given in economic evaluations, and only addressed in six of the eleven reviews.

**Conclusions:**

Economic evaluations of DPHP measures give only little attention to the specific methodological challenges related to this area. For future economic DPHP evaluations a tool with structured guidance should be developed.

This overview of reviews was not registered and a published protocol does not exist.

**Supplementary Information:**

The online version contains supplementary material available at 10.1186/s12889-021-12174-w.

## Background

In light of rapidly ageing populations, disease prevention and health promotion (DPHP) is attracting increasing attention from health policy makers all around the world [1]. According to the World Health Organization (WHO), disease prevention aims to minimize the burden of specific diseases and their associated risk factors by covering population-based and individually focused interventions. Slightly different, health promotion complements these efforts by empowering people to increase their control over determinants of health and developing supportive environments [2].

In 2015, across the member states of the Organisation for Economic Co-operation and Development (OECD), the financial resources spent on DPHP were on average less than 3% of all health spending [1]. An increase of investments in DPHP programs would require conclusive and valid information about the costs and benefits of DPHP-measures. Usually, this information is provided to funding agencies by health economic studies such as cost-effectiveness analyses (CEA, where benefits are expressed in natural effects or physical units), cost utility analyses (CUA, with benefits as health state preference scores), cost-benefit analyses (CBA, monetary terms), cost-minimization analyses (CMA, where only costs are compared) and cost-consequences analyses (CCA with an array of output measures as benefit) [3]. In addition, the social return on investment (SROI) methodology targets broader socio-economic outcomes by analysing views of multiple stakeholders and expressing these in a singular monetary ratio [4].

Although economic evidence for DPHP is increasing [5], health economic studies for DPHP can be affected by significant methodological challenges. Motivated by the Wanless report [6] that described factors likely to have an impact on the resources required to deliver a high-quality health service, the Public Health Research Consortium (PHRC) from the United Kingdom (UK) identified four key elements of economic evaluation in DPHP [7].

(i) the attribution of intervention effects should ensure an adequate reflection of a complex DPHP-measure; since trial-based designs such as randomised controlled trials (RCTs) are not always feasible, economic modelling offers a flexible approach, as it uses multiple data sources [8]. Similarly, the applied data on the consideration of (ii) outcomes and (iii) inter-sectoral costs and consequences (i.e., costs and monetary benefits which spread to other sectors) should reflect the specific context of DPHP. Furthermore, (iv) health equity is one of the main objectives stated in public health policy worldwide [9].

To achieve the social goal of allocating funding more efficiently between health care and public health, it is vital that valid and comparable analytic methods are used for both [6]. Therefore, the objective of this overview was to summarize the above listed key elements for methodological rigor in economic evaluations of DPHP-measures. The evidence was obtained from systematic and scoping reviews of a) previous health economic analyses over a broad range of DPHP areas/measures with b) a methodological focus on the attribution of effects, outcomes, inter-sectoral costs and consequences, or equity. We also discuss the reported challenges in view of published recommendations for economic evaluations of DPHP interventions obtained from various methodological papers.

## Methods

Our overview of reviews was reported according to reporting guidance in the Preferred Reporting Items for Systematic Reviews and Meta-analyses (PRISMA) 2020 reporting guideline statement [10] (see checklist in Additional file 1). Our overview of reviews was neither registered nor a protocol was published.

### Search strategy

A bibliographic search was performed in MEDLINE (via Pubmed), EMBASE (via Elsevier), NHS Economic Evaluation Database (NHS EED), Database of Promoting Health Effectiveness Reviews (DOPHER) and Cochrane Database of Systematic Reviews (CDSR). All search strategies are available in Additional file 2. The websites of the OECD, the ‘Medical Research Council’ and the PHRC and the reference lists of the retrieved articles were also examined (Additional file 3). The search was conducted in September 2020 (updated in October 2021) and neither limits due to publication date nor language were applied.

### Eligibility criteria

We searched for systematic and scoping reviews that provided data on methodological aspects of full health economic DPHP evaluations (i.e. evaluations which compare the costs and effects of two or more alternatives). Scoping reviews differ from systematic reviews in that they provide a broad overview of the evidence on a topic, while systematic reviews are used to address more specific questions based on particular criteria of interest (e.g., population, intervention, outcome) [11]. We included reviews which aimed specifically at the methodological aspects of economic evaluations of DPHP-measures. Reviews were eligible if they i) evaluated full health economic studies addressing evaluations of any DPHP intervention and ii) provide a beforehand stated emphasis on the methodological assessment of the PHRC criteria [7]: (i) the attribution of effects, (ii) the selection of outcomes, (iii) the inclusion of inter-sectoral costs and consequences or, (iv) the consideration of equity aspects in the included studies. That means that, articles which did not prospectively address a methodological focus (via research questions or extraction criteria) were excluded. Reviews that targeted a specific study design (e.g., solely trial-based or model-based evaluation) or study type only (e.g., social return of investment studies), vaccination programs, genomic sequencing, or disease management programs were also excluded due to their specific nature.

### Study selection process

Two reviewers (DM and YS) independently screened the search result (i.e., citations and full-texts) and extracted data from the selected studies using predefined eligibility criteria. Data from the selected studies were extracted by standardized data extraction forms. Differences were resolved through discussion.

### Assessment of methodological quality

We assessed the quality of the reviews using several criteria from the Assessment of Multiple SysTemAtic Reviews tool (AMSTAR) [12], We focused on (1) the study objective, (2) the adequate provision of inclusion criteria, (3) a double-check principle for search and data extraction, (4) details on the literature search, (5) a risk of bias assessment, and (6) a discussion of potential heterogeneity (e.g. differences in modelling approaches, settings, or perspectives). These criteria were chosen because they were assumed to be of particular importance for the quality of the reviews (e.g., addressing meta-analyses). The assessment was undertaken by one reviewer (YS) and cross-checked by another reviewer (DM).

### Data extraction

Based on guidance for evidence synthesis [10, 13], we focused on methodological aspects of economic evaluations targeting DPHP interventions that were likely to affect the study results. Information was selected for the PHRC criteria [7], four main methodological challenges which were derived from evaluating empirical studies of the NHS Economic Evaluation Database (NHS EED). The identified key challenges [7] correspond to a summary of suggestions from key health economics commentators (e.g., Edwards 2013 [14]). To emphasize on DPHP, these specific aspects were preferred to a more general quality assessment. For further assessment, various sub-criteria were selected to reflect the tendency in how researchers deal with specific methodological issues.

#### Attribution of effects: trial versus model based-design, time horizon

According to specific recommendations for economic analyses of DPHP the *time horizon* and an appropriate *study design* has to be chosen [15]. The *time horizon* of the analysis should extend far enough to reflect important differences between the strategies under evaluation. This is of particular interest for interventions where costs and benefits are falling apart (e.g., media campaigns or behavioral measures). A *trial-based* (e.g., a randomised-controlled trial) evaluation examines costs and outcomes within a predefined observation period; a *model-based* evaluation goes beyond this period and either extrapolates costs and outcomes obtained from a trial (e.g., lifetime) or syntheses data from different sources (e.g., clinical trials and routine data).

#### Outcomes: health or preference-based outcomes, intermediate outcomes, non-health outcomes

Intermediate outcomes are commonly used for linking the effect with final health outcomes but causation is often taken into question. For CUAs which are preferred by many decision makers, health outcomes were transferred into *preference-based outcomes* (e.g., quality-adjusted life years (QALYs)). Due to their complex nature, DPHP interventions may also affect wider or *non-health outcomes*, i.e. outcomes that are not covered by dimensions of the EQ-5D: mobility, self-care, usual activities, pain/discomfort and anxiety/depression [16]. On an individual level, non-health-outcomes refer to self-confidence, insights into one’s own (un) healthy behavior, and perceived life control [17] which can be used as a single measure or be included in wider conceptualizations of quality of life or wellbeing.

#### Inter-sectoral costs and consequences: perspective and cost categories

A major challenge in DPHP is to include inter-sectoral costs and consequences, i.e. *categories* of costs that accrue to non-health sectors of society and could inform CBAs. Based on Drost et al. we assessed the cost categories ‘education’ (e.g., learning aids), ‘labor and social security’ (e.g., productivity losses due to absence from work or costs due to reintegration after imprisonment), ‘household and leisure’ (e.g., informal care), ‘criminal justice’ (e.g., police interventions) and, ‘other individual and family effects’ (e.g., family conflicts). These costs may contribute a considerable amount to the total costs and benefits of interventions in the health care sector [18]. The perspective taken for an analysis determines the extent to that inter-sectoral costs and consequences will be considered. For example, an economic study of a DPHP-measure to prevent substance abuse rarely considered criminal justice costs if the study was funded by the healthcare sector [19].

#### Equity: consideration and method of inclusion

In response to policy concerns regarding health equity, economic evaluation of DPHP should provide useful evidence on health equity impacts. Who gains and who loses out from a health program depends individual health risks, uptake of and adherence to measures, capacity to benefit, and—crucially—who bears the opportunity costs of diverting scarce resources from other uses [20].

### Data synthesis

Because the quantitative information obtainable from the reviews was limited, a narrative synthesis without a meta-analysis or other statistical methods was conducted [21, 22] In particular, to summarize the information about the methodological aspects we tabulated [13] both the qualitative (e.g. scope of the analysis or types of outcomes) and the quantitative data (e.g. frequencies of corresponding outcomes) for each review included. Further, based on the reported data in the included reviews, we estimated the weighted proportion of studies meeting the sub-criteria of extracted data across the included reviews and presented the results visually using bar charts.

## Results

### Reviews identified using the search

**Fig. 1:**
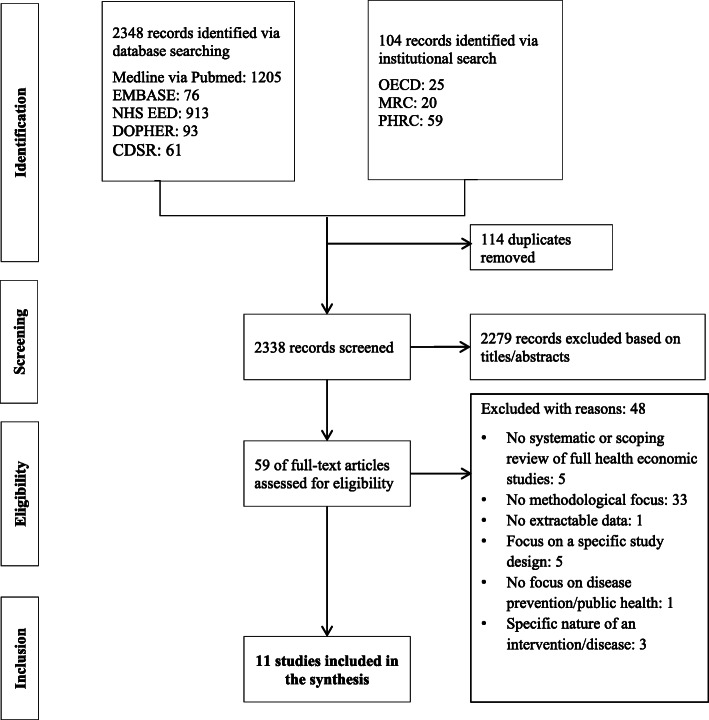
Flow diagram of the search. OECD, Organisation for Economic Cooperation and Development; MRC, Medical Research Council; PHRC, Public Health Research Consortium

The search returned 2452 records. After screening titles and abstracts, 59 records were examined for potential eligibility. The inclusion criteria assessment resulted in ten systematic and one empirical (scoping) review (Fig. 1) [5, 19, 23–31] Excluded reviews are shown in Additional file 4. Of the included reviews (Table 1), five addressed different preventive measures for older people [25, 27] or children/adolescents [26, 28, 30], while the others targeted physical activity [23], implementation of DPHP [24], alcohol prevention [31], behavior change [5], injury prevention [29], or different areas of prevention [19]. Overall, these reviews included 494 economic analyses (CEA: 42%, CUA: 20%, different types of economic study: 16%, CCA: 15%, CBA: 6%, CMA: 1%. SROI: 0,4%).

**Table 1:** Summary of methodological aspects reported in reviews of health economic analyses

Source	Area / Study type	Attribution of effects	Outcomes	Inter-sectoral costs	Equity
Cochrane et al. (2019) [23]	Physical activity and sedentary behaviour interventions *n* = 15 CEA = 40% CUA = 33% Different types = 27%	**TBA/MBA:** *n* **=** 10/5 **Time horizon all**: - ≤ 2y: 67% - lifetime: 33% **TBA:** ≤ 2y 100% **MBA:** lifetime 100%	**Intermediate HO**: 53% **HO/PbO**: 60% (QALYs) **Non-HO**: 13% (e.g., absenteeism)	**Categories:** **-** labor/soc. security: 20% **Perspective**: - societal: 27% - health sector: 47% - not stated: 13%	Implicitly included via subgroup analyses: 93% (e.g., targeting the intervention to individuals in need of care)
Reeves et al. (2019) [24]	Implementation of DPHP-interventions *n* = 14 CEA = 86% CUA = 7% CBA = 7%	**TBA/MBA:** *n* **=** 12/2 **Time horizon all**: - ≤ 2y: 43% - lifetime: 0% - not reported: 36% **TBA**: ≤ 2y 50% **MBA**: lifetime 0%	**Intermediate HO**: 93% **HO/PbO**: 29% (QALYS, life saved) **Non-HO**: 7% (violent crimes)	**Categories:** - education: 43% - labor/soc. security: 29% - criminal justice: 7% **Perspective**: - societal: 42% - not stated: 21%	n.a.
Zanganeh et al. (2019) [30]	Childhood and adolescent obesity interventions *n* = 28 CEA = 36% CUA = 32% CCA = 4% Different types = 29%	**TBA/MBA:** *n* **=** 9/19 **Time horizon all**: - ≤ 2y: 21% - lifetime: 39% - not reported: 7% **TBA**: ≤ 2y 67% **MBA**: lifetime 58%	**Intermediate HO**: all studies (64%: BMI) **HO/PbO**: 59% (QALYs, DALYs) **Non-HO**: n.a.	**Categories**: - education: 4% - labor/soc. security: 57% - other: 18% **Perspective**: - societal: 84% - health sector: 8% - not stated: 8%	n.a.
Huter et al. (2018) [25]^a^	DPHP-interventions for older people *n* = 8 CEA = 25% CUA = 25% Different types = 50%	**TBA/MBA:** *n* = 4/4 **Time horizon all**: - ≤ 2y: 75% - lifetime: 13% **TBA**: ≤ 2y 100% **MBA**: lifetime 25%	**Intermediate HO**: all studies (e.g., falls, physical activity) **HO/PbO**: 88% (fall-related fractures, QALYs) **Non-HO**: 13% (social benefits such as ‘general self-efficacy’, ‘well-being’, or ‘loneliness’)	**Categories**: − labor/soc. security: n.a. − household/leisure: n.a. − other: 13% **Perspective**: - societal: 13% - not stated: 88%	No adjustment for the preference structure of elderly in CUAs
Oosterhoff et al. (2018) [26]	School-based lifestyle interventions *n* = 23 CEA = 48% CUA = 17% CBA = 9% SROI = 4% Different types = 22%	**TBA/MBA:** *n* = 9/14 **Time horizon all:** - ≤1y: 30% - lifetime: 43% **TBA**: ≤1y 78% **MBA**: lifetime 71%	**Intermediate HO:** 48% (weight-related measures or BMI) **HO/PbO:** 39% (QALYs or DALYs) **Non-HO:** 13% (school behaviour, wellbeing, outcomes to the household and leisure sector, environmental impacts and externalities, only in CBAs, SROI)	**Categories**: - education: 9% - labor/soc. security: 17% - household/leisure: 13% - other: 4% **Perspective:** **-** societal: 74% - healthcare: 13% - programs perspective: 4% - not stated: 9%	n.a.
Dubas-Jakóbczyk et al. (2017) [27]	DPHP-interventions for older people *N* = 29 CCA = 7% CEA = 45% CUA = 10% CBA = 10% Different types = 28%	**TBA/MBA:** *n* = 19/10 **Time horizon all**: - ≤ 1y: 50% - lifetime: 17% **TBA**: ≤ 1y n.s **MBA**: lifetime n.s	**Intermediate HO**: 35% (falls, number of falls or fallers prevented) **HO/PbO**: 65% (changes in SF-36 physical functioning, LYG, daily functioning changes, QALYs) **Non HO**: 3% (e.g., social network)	**Categories**: - labor/soc. security: 3% - household/leisure: 21% - other: 7% **Perspective**: - societal: 41% - payer: 52% - unclear: 7%	Lack of economic evaluations for the prevention of mental health problems among the population 65+
Hill et al. (2017) [31]	Alcohol prevention *n* = 27 CEA = 22% CUA = 48% CBA = 4% CCA = 4% SROI = 4% Different types = 19%	**TBA/MBA:** *n* = 8/19 **Time horizon all:** - ≤1y: 19% − 1-5y: 26% - >30y to lifetime: 37% - not reported: 11% **TBA**: - ≤1y 50% **MBA**: - >30y to lifetime 53%	**Intermediate HO**: n.a **HO/PbO:** 67% (e.g., QALYs) **Non HO**: 0%	**Categories:** - education: 11% - labor/soc. security: 26% **-** household/leisure: 15% - criminal justice: 33% **Perspective:** **-** societal: 52% - health sector: 41% - other or not stated: 7%	Subgroup analysis for gender, age and alcohol intake
Döring et al. (2016) [28]	Obesity prevention in early childhood *N* = 6 CCA = 17% CEA = 83%	**TBA/MBA**: *n* = 2/4 **Time horizon all**: - ≤2y: 33% - lifetime: 67% **TBA**: ≤2y 100% **MBA**: lifetime 100%	**Intermediate HO**: 100% (BMI, weight, behavioral changes) **HO/PbO**: 33% **Non HO**: n.a.	**Categories**: - labor/soc. security: 67% **Perspective**: - societal: 50% - other: 17%	n.a.
Alayli-Goebbels et al. (2014) [5]	Behavior change interventions *N* = 142 CCA = 8% CEA = 48% CUA = 11% CMA = 3% CBA = 6% Different types = 25%	**TBA/MBA**: *n* = 58/56^b^ **Time horizon all:** - ≤ 2y: 37% -lifetime: 21% - not reported: 22% **TBA**: ≤ 2y 83% **MBA**: lifetime in 38% (30/79 modelling and combined)	**Intermediate HO**: 61%: (behavior change, biomedical health indicators) **HO/PbO**: 52% (e.g., survival, HRQOL) **Non HO**: 9% (e.g., increased health knowledge)	**Categories**: - summarized: 8% (included costs of car accidents, violent crimes, personal injury, property damage, fire destruction, law enforcement, and costs to industry, commerce and the voluntary sectors) **Perspective**: - not stated: 43%	Explicitly considered by no study
Polinder et al.(2012) [29]	Injury prevention *N* = 48 CEA = 38% CUA = 10% CBA = 35% Different types = 17%	**TBA/MBA**: *n* = 24/24 **Time horizon all**: - ≤ 5y: 33% - lifetime: 8% - not reported: 17% **TBA**: <2y 17% **MBA**: lifetime 16%	**Intermediate HO**: 10% (falls prevented) **HO/PbO**: 52% (injuries prevented, life saved, QALYs) **Non HO**: n.a.	**Categories**: - labor/soc. security: 23% - household/leisure: 2% **Perspective**: - societal: 69% - health care: 25%	n.a.
Weatherly et al. (2009) [19]	DPHP-interventions in eleven public health areas *N* = 154 CCA = 37% CEA = 36% CUA = 27% CMA = 0% CBA = 0%	**TBA/MBA**: *n* = 106/48 **Time horizon all**: n.s.	**Intermediate HO**: n.s. (falls prevented, pounds lost) **HO/PbO**: 27% (QALYs, DALYs) **Non HO:** n. s. (quality of wellbeing, public preferences for the (dis) benefits of a water fluoridation program)	**Categories:** - education: 3% **-** labor/soc. security: 20% - household/leisure: 2% - criminal justice: 4% - other: 16% **Perspective**: - payer: 32% - societal: 31% - not stated: 24%	QALYs were not explicitly equity-weighted, some studies conducted equity-related sub-groups analyses

*BMI* body mass index, *CCA* cost-consequences analysis, *CBA* cost-benefit analysis, *CEA* cost-effectiveness analysis, *CMA* cost minimization analysis, *CUA* cost-utility analysis, *EE* economic evaluation, *HO* health outcome, *N* number, *n.a* not addressed, *n.s* proportion or details not specified, *MBA* model-based analysis, *PbO* Preference-based outcome, *TBA* = trial-based analysis, *QALY* quality-adjusted life years, *DALYs* disability-adjusted life years, *HRQOL* health-related quality of life, *SROI* social return on investment, *WTP* willingness to pay, *y* year(s)

^a^ Eight of 37 studies included because other studies were already included in Dubas-Jakóbczyk et al. [27]

^b^ Remaining studies were based on a combination of TBA and MBA or, the design was not described (3.5%)

#### Attribution of effects

In 44% of the individual studies the time horizon was less than 5 years, while a lifelong time horizon was adopted in about 23% of the analyses. Trial-based approaches were used more often than model-based approaches to evaluate DPHP interventions (56% vs. 44%), while efforts to synthesize trial data with a modelling approach were found in 10% of DPHP evaluations. With regard to trial-based analyses, 64% had a time horizon up to 2 years [5, 23–26, 28–31].

Overall, the research designs varied widely and were insufficiently reported. For example, comparators under evaluation were not described clearly [19, 26], costs and effects were often not discounted to adjust for different timing [5, 29] or - in model-based studies - the model choice or the underpinning assumptions (e.g., maintenance of effects over time) were not explained clearly or justified sufficiently [23, 28, 30]. Similarly, heterogeneous methods were used in both model and trial-based studies to extrapolate intermediate to final outcomes (e.g., change in BMI and its influence on morbidity), to extend the timeframe of the analysis (e.g., extrapolating the impact on future health-related outcomes), and/or to synthesize data from different sources (e.g., decision-analytic model or regression model) [19, 26].

#### Outcomes

In 56% of individual studies, researchers relied on intermediate health outcomes such as biomedical health indicators (e.g., weight loss) [5, 19], falls as an intermediate for fractures [19, 27], or indicators related to the avoidance of health service use (e.g., hospital admissions prevented) [5, 27]. Many studies with a long-time horizon did not account for the possibility of diminishing intervention effects [26].

Whereas measures of (direct) health outcomes such as life years gained were used rarely, preference-based outcomes were applied in about 36% of the individual studies. Preferences were expressed by using QALYs or disability-adjusted life years (DALYs) and, by converting a statistical life into monetary values resulting in a CBA. Moreover, contingent valuation willingness-to-pay (WTP) surveys were used for eliciting public preferences [7, 19].

Eight percent of the economic studies measured non-health outcomes or used broader definitions of quality of life or wellbeing (e.g., measures of health literacy, improvement of self-management capacities, and social networks). For example, two studies (1.4%) used measures of global quality of life or wellbeing in evaluations of behavior change interventions [5]. Furthermore, outcomes for individuals not directly targeted by the intervention and community-level outcomes were rarely incorporated when evaluating an intervention [5, 26].

#### Inter-sectoral costs and consequences

The costs of labor/social security (i.e., productivity losses due to absence from work) were considered in 23% of the individual studies. In contrast, the costs of education (7%), household & leisure (6%) and criminal justice (8%) were included only in a small number of studies. Other monetary individual/family effects were included in 14% of the studies. Compared to reviews published before 2017 [5, 19, 28, 29], in more recent reviews there was a slight increase in the consideration of inter-sectoral costs and consequences.

The reviews raised concerns about an often incomplete reporting of both health sectoral costs and inter-sectoral costs. Moreover, several reviews addressed inconsistencies in the cost analysis process, such as the usage of different approaches to classify, measure and value the same cost categories [5, 24, 25, 27].

According to the included reviews, the underlying economic studies showed often inconsistencies between the chosen perspective and the inter-sectoral costs considered. For example, although 46% of all studies applied a societal perspective, the costs due to labor/social security were calculated for only half of these studies. Furthermore, many studies failed to state the perspective of the analysis [5, 19, 23, 24]. In some studies, the perspective was indicated without specifying the cost categories considered [26] or the range of costs and consequences was narrower than expected for the stated perspective [23].

#### Equity

While concerns of equity were not addressed in almost half of the reviews [24, 26, 28–30], the authors of five reviews concluded that equity considerations were usually disregarded in economic studies [5, 19, 25, 27, 31]. In particular, Alayli-Goebbels et al. (2014) [5] noted that the impact on equity was not examined at all, neither by using equity-weighted utilities nor by calculating cost-effectiveness ratios for different socio-economic groups. Although some studies provided calculations for sub-groups that might be relevant for equity considerations, these studies usually summarized utilities, implying equal weighting no matter to whom the benefits accrue [19]. Only the most recent review noted that almost all of the included analyses considered equity. However, the findings in this review suggest that analysts are not performing equity analyses in a comprehensive or consistent manner [23]. For example, only one of 15 studies in this review researched socio-economic status by asking participants about their WTP for an intervention component.

**Fig. 2:**
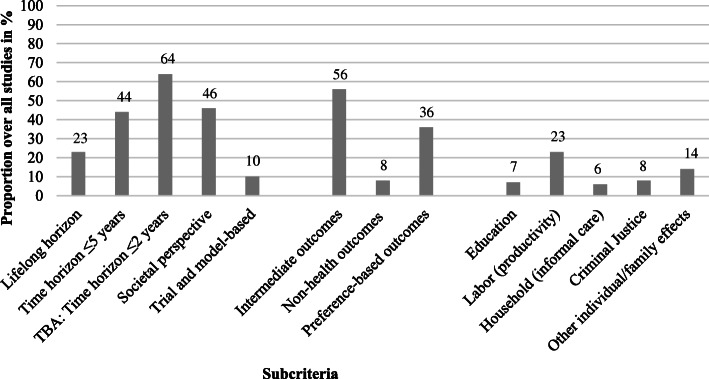
Proportion of relevant subcategories reported in the included reviews (weighted)

Figure 2 provides an overview of the overarching analyses conducted to summarize evidence on methodological approaches in the underlying studies for each of the four PHRC criteria (Fig. 2 and Additional file 6)

### Methodological quality of the reviews

Based on several criteria of the AMSTAR-tool, the methodological quality of the reviews was sufficient [12]. All reviews provided information on their objective, inclusion criteria, literature search, and study and data extraction methods. The quality of the underlying economic studies was addressed in 9 reviews [5, 23–30] while heterogeneity between the included analyses was addressed in seven [24–30]. Reeves (2019) [24] and Alayli-Goebbels (2014) [5] explicitly evaluated the methodological quality of the underlying studies. Moreover, Reeves (2019) [24] differentiated between the methodological and reporting quality of the included economic analyses (see Additional file 5).

## Discussion

Our comprehensive overview summarizes how economic evaluations of DPHP-measures considered methodological challenges of economic evaluation in DPHP. According to eleven reviews (494 analyses), there were methodological inconsistencies over a broad range of DPHP measures for all predefined dimensions (attribution of effects, quantification of outcomes and costs, consideration of equity). Whereas current theoretical debates (e.g., [9, 14, 18, 32, 33]) of specific challenges in economic evaluations of DPHP are rarely reflected in health evaluation practice, non-compliance with well-established general standards of economic evaluation [34] was also often observed. As a result, the information obtained from many health economic analyses of DPHP measures may be limited in value for decision maker. Encouragingly, recent reviews [23, 24, 30] indicate a tendency to address the specific challenges of DPHP more appropriately (e.g., inter-sectoral costs and consequences, equity).

To account for the *attribution of effects*, the increased number of relationships and interactions in DPHP-models (e.g., between behavior changes, biomedical health indicators, patients, care giver) requires the application of models with more flexibility than conventional Markov-models (e.g., [5, 25, 28, 31]). Because the cost-effectiveness of a DPHP measure is predominantly determined by individual behavior, some authors proposed more sophisticated modelling techniques such as econometric modeling, individual-level Markov models, discrete event simulation (DES), social network analysis or agent-based simulation (ABS) [5, 19].

More specifically, a conceptual modelling approach as recommended by Squires et al. 2016 can support the development of model structures that reflect the dynamic and complex nature of DPHP interventions [32]. Conceptual modelling allows to consider data on the uptake of an intervention, unforeseen participant responses (e.g., non-participation), or variations in the provision of measures (e.g., frequency or the care-giver involved). To reflect a complex and dynamic DPHP-measure, conceptual modeling framework refers to a holistic way of thinking about the interactions between parts within a system and with its environment [35]. By defining multiple system levels which are subjectively defined, higher level systems correspond to lower level systems reflecting more detailed aspects [32].

Moreover, conceptual models are assumed to allow the consideration of various determinants of health [36] (e.g., the social, economic, and physical environment, as well as a person’s individual characteristics). By including broader determinants of health, conceptual models can also be used to facilitate identification of non-health costs and outcomes associated with the DPHP-measures [32].

When preferring a trial-based approach, cluster-randomised community trials or registry-based RCTs (i.e., pragmatic trials that use registries for data collection, randomisation, and follow-up) may prove an appropriate tool for comparative effectiveness in real-world settings [37]. However, the majority of trial-based evaluations are based on short time horizons (e.g. [23]), indicating that decision analytic modelling should be used more often (to date: 10%) for extrapolating the findings of a study beyond the period of observation (e.g., [5, 23, 26]).

With regard to the *outcomes* used for evaluations of DPHP, authors of the reviews reported both a lack of usage of non-health outcomes and a lack of valuing outcomes. According to the reviews, intermediate outcomes were predominantly used in health economic analyses of DPHP interventions (56%), while non-health outcomes and preference-based outcomes are rarely applied (8 and 36%, respectively), resulting in a broad consensus for increasing their consideration [5, 25–27]. Based on current methodological guidance, some researchers argue for the inclusion of non-health outcomes in a tool [14, 38, 39] but disagree on what is more promising: the development of a new tool, or the use of established questionnaires with a theoretical founding (e.g., QALYs).

Over 60% of the studies in the included reviews did not attempt any outcome valuation, i.e. they provide a CCA (15%) or CEA (42%). In contrast, 20% of the studies were preference-based CUAs, with valuations restricted to health outcomes expressed in QALYs or DALYs. A CUA is grounded in a non-welfarist approach, while other studies with outcome valuation were based on a CBA (6%), a welfarism-approach with outcomes valuated in monetary terms using willingness-to-pay estimates or SROI. A CBA might be useful for adapting the analysis to the relevant outcomes beyond health [31]; however, practical issues associated with monetary valuation remain unresolved [32]. Given the ongoing discussion on how to measure and value non-health outcomes, a CCA appears to be useful for providing a disaggregated overview of DPHP interventions [19, 32].

For the future, available methods should increasingly be used for the valuation of non-health outcomes. These include ‘contingent valuation’ [5], ‘willingness to pay’ for eliciting a benefit of an intervention [5, 19], and ‘discrete choice techniques’ [40]. In addition, outcomes caused by externalities (i.e. outcomes for individuals who are not directly targeted by the intervention) should be included more often [5].

Because *inter-sectoral costs and consequences* were excluded in economic studies of DPHP for a long time [5, 19, 28, 29], several researchers highlighted the need to consider these costs [5, 14, 19, 32, 41]. However, the tools developed were heterogeneous and showed limited evidence on validity and reliability [42].

In 2013, Drost et al. developed and applied several approaches for the inclusion of impacts for the education and criminal justice sectors [18], resulting in a slight increase of reporting inter-sectoral costs and consequences in later reviews. However, a prerequisite is that data valuation can be based on proxy unit prices, on self-constructed unit prices or on labor costs [18].

The consideration of labor costs, even in evaluations adopting a societal perspective, is controversial for different reasons. First, the inclusion of labor costs is contentious and, there is no consensus or guidance on how to measure and value productivity appropriately, in particular with regard to unpaid work. Second, productivity costs are often disregarded because they are assumed to be negligible. However, the exclusion of productivity costs may result in underestimating the cost-effectiveness of DPHP measures [25].

DPHP interventions can be evaluated from a number of different perspectives, e.g., the health sector perspective, the public sector perspective or the perspective of particular agencies involved in the system [32]. The failure to consistently adopt a societal perspective (46% and, in studies with a societal perspective, the omission of certain relevant costs such as those relating to productivity losses and participants’ time, may underestimate the cost-effectiveness of DPHP-measures [43] and often precludes a deeper understanding of the monetary consequences of DPHP.

Although validated and well-accepted tools for the inclusion of these inter-sectoral costs are lacking, the adoption and consistent application of a societal perspective would stimulate efforts to include costs and effects beyond the health sector [42].

Although many authors have called for the incorporation of *equity concerns* in economic evaluations in DPHP, existing methods [9, 38, 44] are neither common practice nor included in guidelines for economic evaluations. As a result, most evaluations in DPHP fail to consider equity. At least, the most recent review observed an increased consideration of equity aspects by conducting subgroup analyses or targeting a population deemed in need of intervention [23]. This might indicate growing awareness of this issue.

In general, equity considerations might be qualitatively examined by providing background information on aspects of fairness [5, 19, 45]. A more extensive approach aims to calculate cost-effectiveness for different ‘equity-relevant’ subgroups [9] characterized by socio-economic status, geographical location or ethnicity [5, 19, 23, 45]. A third approach aims to estimate the opportunity cost of a particular equity consideration in terms of population health sacrifice. For example, the life years lost by pursuing a more equitable option can be opposed to a more health maximizing option. The resulting opportunity cost is an approximate for the monetary value of considering equity [9, 20].

The equity weighting analysis approach requires more information. This method aims to explicitly value the reduction of health inequality [19, 45]. This analysis quantifies the trade-offs between improving total health and other equity objectives. The idea of this approach is to weight health gains (e.g., life years) with different equity-relevant characteristics. The weights are based on values elicited from specific stakeholders such as the general public or/and policy makers. The weights can be elicited using common health outcome valuation techniques (e.g., discrete choice experiments) [9, 20] Weatherly et al. (2009) emphasize that more research is needed on equity-weighting issues for an individual’s social-economic status, personal responsibility for health risks, and the preference toward treating current illnesses versus preventing future health risk [19]. Alternatively, equity might be considered by Sen’s capability theory [46] which accounts for the distribution of capability across society.

### Strengths and limitations of this overview

By taking up the quality criteria for economic evaluations identified by the PHRC, specific methodological challenges could be observed for predefined key elements of DPHP-interventions. Based on these categories, the results of our overview provide a comprehensive picture of the challenges for economic evaluations in DPHP. Thus, this analysis may provide a starting point for developing structured guidance for conducting economic evaluations in DPHP.

Although the methods used for this analysis were in line with the principles of good systematic reviewing [10, 12, 13] some limitations are inherent: first, the overview was limited to studies focusing on methodological aspects. As a result, we did not include methodological challenges from systematic or scoping reviews of economic studies without this focus.

Second, in the reviews methodological aspects were heterogeneously reported. Furthermore, data on sub-criteria to be extracted was not always provided (or extractable), resulting in only approximate estimates and the impossibility to evaluate interrelationships between these sub-criteria (e.g., to calculate the proportion of studies with a societal perspective that included productivity costs). Therefore, this overview presents a trend in the modus operandi for DPHP evaluations.

Third, there may be other dimensions and sub-dimensions reflecting relevant aspects of the validity of economic DPHP evaluations which were not addressed in the PHCR-criteria (and thus not in our overview). Among others, these may include dealing with uncertainty, the choice of discount rates, and the inclusion of costs due to screening or costs resulting from added life years. However, by relying on the PHCR-criteria, we focused on dimensions of quality that were considered to be essential in the context of DPHP [7].

Fourth, the specific relevance of each of the challenges (i) to (iv) in an economic study depend on several factors such as the specific interventions under evaluation, the target group, the objective, the chosen study type or the perspective. As a result, the need to make particular efforts in the methodological key elements may have differed between the studies. Because we included evaluations of any DPHP-measure, the differences requiring to address the specific challenges more or less were not considered in this overview.

Finally, some economic evaluations might have been included in different reviews. Since not all reviews reported the individual studies they included, we cannot rule out that double-counting occurred (Fig. 2). However, we believe that the main conclusions drawn from this analysis would not alter.

## Conclusion

Economic evaluations of DPHP measures often disregard the specific methodological challenges related to this area. Future research should aim to develop structured guidance (including a checklist) that provides recommendations for researchers who intend to economically evaluate a DPHP measure. Ideally, this tool should combine the basic principles of health economic evaluation with the particular challenges relating to DPHP. For this purpose, we recommend establishing an expert task force to identify (i) which aspects of already established guidelines may correspond to DPHP and, (ii) which issues require further specifications or methodological advancement. Additionally, there is a strong need for an increased dialogue between different stakeholders (e.g. decision-makers and academia) in order to scrutinize the boundaries of economic analyses in the field of DPHP.

## Supplementary Information

**Additional file 1.** PRISMA 2020 Checklist.

**Additional file 2.** Electronic Database Searches.

**Additional file 3.** Institutions.

**Additional file 4.** List of the excluded studies.

**Additional file 5.** Assessment of methodological aspects of the reviews included.

**Additional file 6.** Quantitative Synthesis.

## Data Availability

The datasets used and/or analysed during the current study are available from the corresponding author on reasonable request.

## Abbreviations

AMSTAR: Assessment of Multiple SysTemAtic Reviews
ABS: Agent-based simulation
CBA: Cost-benefit analysis
CCA: Cost-consequences analysis
CDSR: Cochrane Database of Systematic Reviews
CEA: Cost-effectiveness analysis
CHAP: Cardiovascular Health Awareness Program
CMA: Cost-minimization analysis
CUA: Cost-utility analysis
DALY: Disability-adjusted Life Year
DES: Discrete Event Simulation
DPHP: Disease Prevention and Health Promotion
DOPHER: Database of Promoting Health Effectiveness Reviews
ICECAP-A: Investigating Choice Experiments Capability Measure for Adults
NNS EED: NHS Economic Evaluation Database
OECD: Organisation for Economic Co-operation and Development
PHRC: Public Health Research Consortium
QALY: Quality-adjusted Life Year
RCT: Randomised controlled trial
SROI: Social return on investment
UK: United Kingdom
WTP: Willingness-to-pay
