# Supplementary material for: Methodological issues in economic evaluations of disease prevention and health promotion: an overview of systematic and scoping reviews
Source: BMC Public Health. 2021 Nov 20;21:2130. doi: 10.1186/s12889-021-12174-w (PMC8605499; doi:10.1186/s12889-021-12174-w)
Supplement: Supplementary file 2 — Additional file 2. Electronic Database Searches. [file 12889_2021_12174_MOESM2_ESM.docx]

# Additional file 2:

Electronic Database Searches

| Database | Terms |
| --- | --- |
| MEDLINE via Pubmed | #1 Methods[Mesh]  #2 method*[TIAB]  #3 costs and cost analysis[MeSH]  #4 health economics[TIAB]  #5 econ* evaluation*[TIAB]  #6 Preventive Health Services[MeSH]  #7 prevention program[TIAB]  #8 health promotion[TIAB]  #9 public health[TIAB]  #10 public health intervention*[TIAB]  #11 systematic review[TIAB]  #12 scoping review[TIAB]  #13 empirical review[TIAB]  #14 (#1 OR #2)  #15 (#3 OR #4 OR #5)  #16 (#6 OR #7 OR #8 OR #9 OR #10)  #17 (#11 OR #12 OR #13)  #18 (#14 AND #15 AND #16 AND #17) |
| NHS EED | #1 MeSH DESCRIPTOR Methods EXPLODE ALL TREES IN NHSEED  #2 (MeSH Descriptor Costs and cost Analysis Explode all trees) IN NHSEED  #3 (MeSH Descriptor Preventive Health Services Explode all trees) IN NHSEED  #4 (method*) IN NHSEED  #5 (health economics) IN NHSEED  #6 (econ* eval*) IN NHSEED  #7 (cost-effectiveness) IN NHSEED  #8 (prevention program) IN NHSEED  #9 (health promotion) IN NHSEED  #10 (public health) IN NHSEED  #11 (public health intervention) IN NHSEED  #12 (scoping review) IN NHSEED  #13 (systematic review) IN NHSEED  #14 (#2 OR #5 OR #6 OR #7)  #15 (#3 OR #8 OR #9 OR #10 OR #11)  #16 (#12 OR #13)  #17 (#1 OR #4)  #18 (#14 AND #15 AND #16 AND #17) |
| DoPHER | #1 Freetext (All but Authors): "method*"  #2 Freetext (All but Authors): "health econom*"  #3 Freetext (All but Authors): "cost effectiveness"  #4 Freetext (All but Authors): "economic eval*"  #5 Freetext (All but Authors): "prevention program"  #6 Freetext (All but Authors): "health promotion"  #7 Freetext (All but Authors): "public health"  #8 Freetext (All but Authors): "public health intervention"  #9 (#2 OR #3 OR #4)  #10 (#5 OR #6 OR #7 OR #8)  #11 (#1 AND #9 AND #10) |
| CDSR | #1 MeSH decriptor: [Methods] explode all trees  #2 ("method*"):ti,ab,kw  #3 MeSH descriptor: [Costs and Cost Analysis] explode all trees  #4 ("cost-effectiveness"):ti,ab,kw  #5 ("economic evaluation"):ti,ab,kw  #6 ("health econom*"):ti,ab,kw  #7 ("econ* eval*"):ti,ab,kw  #8 ("prevention program"):ti,ab,kw  #9 ("prevention"):ti,ab,kw  #10 ("health promotion"):ti,ab,kw  #11 ("public health"):ti,ab,kw  #12 MeSH descriptor: [Public Health] explode all trees  #13 (#1 OR #2)  #14 (#3 OR #4 OR #5 OR #6 OR #7)  #15 (#8 OR #9 OR #10 OR #11 OR #12)  #16 (#13 AND #14 AND #15) |
| EMBASE via Elsevier | #1 method*: ab,ti  #2 'methodology'/exp  #3 'health economics'/exp  #4 'health economics':ab,ti  #5 'econ* evaluation*':ab,ti  #6 'cost-effectiveness':ti,ab  #7 'preventive health service'/exp  #8 'prevention program':ab,ti  #9 'health promotion':ab,ti  #10 'public health':ab,ti  #11 'public health intervention*':ab,ti  #12 'systematic review':ab,ti  #13 'scoping review':ab,ti  #14 (#1 OR #2)  #15 (#3 OR #4 OR #5 OR #6)  #16 (#7 OR #8 OR #9 OR #10 OR #11)  #17 (#12 OR #13)  #18 (#14 AND #15 AND #16 AND #17)  #19 (#18 AND [embase]/lim NOT ([embase]/lim AND [medline]/lim))  #20 (#19 NOT ('conference abstract'/it OR 'conference paper'/it OR 'conference review'/it)) |
