# Supplementary material for: Methodological issues in economic evaluations of disease prevention and health promotion: an overview of systematic and scoping reviews
Source: BMC Public Health. 2021 Nov 20;21:2130. doi: 10.1186/s12889-021-12174-w (PMC8605499; doi:10.1186/s12889-021-12174-w)
Supplement: Supplementary file 3 — Additional file 3. Institutions. [file 12889_2021_12174_MOESM3_ESM.docx]

# Additional File 3:

Institutions

| 1. Organisation for Economic Co-operation and Development, OECD   (<http://www.oecd.org/>)   1. Public Health Research Consortium   (<http://phrc.lshtm.ac.uk/>)   1. Medical Research Council   (<https://www.mrc.ac.uk/>) |
| --- |
