# Supplementary material for: Methodological issues in economic evaluations of disease prevention and health promotion: an overview of systematic and scoping reviews
Source: BMC Public Health. 2021 Nov 20;21:2130. doi: 10.1186/s12889-021-12174-w (PMC8605499; doi:10.1186/s12889-021-12174-w)
Supplement: Supplementary file 4 — Additional file 4. List of the excluded studies. [file 12889_2021_12174_MOESM4_ESM.docx]

# Additional File 4:

List of the excluded studies

| Nr. | Reference | Reason for exclusion |
| --- | --- | --- |
| 1. | Schwappach DL, Boluarte TA, Suhrcke M. The economics of primary prevention of cardiovascular disease - a systematic review of economic evaluations. Cost Eff Resour Alloc. 2007;5:5. | B |
| 2. | Lutz N, Clarys P, Koenig I, Deliens T, Taeymans J, Verhaeghe N. Health economic evaluations of interventions to increase physical activity and decrease sedentary behavior at the workplace: a systematic review. Scand J Work Environ Health. 2020;46(2):127-142. | B |
| 3. | Mattli R, Farcher R, Syleouni ME, et al. Physical Activity Interventions for Primary Prevention in Adults: A Systematic Review of Randomized Controlled Trial-Based Economic Evaluations. Sports Med. 2020;50(4):731-750. | B |
| 4. | Bardach AE, Alcaraz AO, Ciapponi A, et al. Alcohol consumption's attributable disease burden and cost-effectiveness of targeted public health interventions: a systematic review of mathematical models. BMC Public Health. 2019;19(1):1378. | D |
| 5. | Lutz N, Taeymans J, Ballmer C, Verhaeghe N, Clarys P, Deliens T. Cost-effectiveness and cost-benefit of worksite health promotion programs in Europe: a systematic review. Eur J Public Health. 2019;29(2):380. | B |
| 6. | Schmidt M, Werbrouck A, Verhaeghe N, et al. Strategies for newborn screening for cystic fibrosis: A systematic review of health economic evaluations. J Cyst Fibros. 2018;17(3):306-315. | F |
| 7. | Roberts S, Barry E, Craig D, Airoldi M, Bevan G, Greenhalgh T. Preventing type 2 diabetes: systematic review of studies of cost-effectiveness of lifestyle programmes and metformin, with and without screening, for pre-diabetes. BMJ Open. 2017;7(11):e017184. | B |
| 8. | Bustamante Madsen L, Eddleston M, Schultz Hansen K, Konradsen F. Quality Assessment of Economic Evaluations of Suicide and Self-Harm Interventions. Crisis. 2018;39(2):82-95. | B |
| 9. | Lal A, Moodie M, Peeters A, Carter R. Inclusion of equity in economic analyses of public health policies: systematic review and future directions. Aust N Z J Public Health. 2018;42(2):207-213. | F |
| 10. | Duncan KM, MacGillivray S, Renfrew MJ. Costs and savings of parenting interventions: results of a systematic review. Child Care Health Dev. 2017;43(6):797-811. | A |
| 11. | Lian JX, McGhee SM, Chau J, Wong CKH, Lam CLK, Wong WCW. Systematic review on the cost-effectiveness of self-management education programme for type 2 diabetes mellitus. Diabetes Res Clin Pract. 2017;127:21-34. | B |
| 12. | Nkonki L, Tugendhaft A, Hofman K. A systematic review of economic evaluations of CHW interventions aimed at improving child health outcomes. Hum Resour Health. 2017;15(1):19. | B |
| 13. | Teljeur C, Moran PS, Walshe S, et al. Economic evaluation of chronic disease self-management for people with diabetes: a systematic review. Diabet Med. 2017;34(8):1040-1049. | B |
| 14. | Flego A, Dowsey MM, Choong PF, Moodie M. Addressing obesity in the management of knee and hip osteoarthritis - weighing in from an economic perspective. BMC Musculoskelet Disord. 2016;17:233. | B |
| 15. | McKinnon RA, Siddiqi SM, Chaloupka FJ, Mancino L, Prasad K. Obesity-Related Policy/Environmental Interventions: A Systematic Review of Economic Analyses. Am J Prev Med. 2016;50(4):543-549. | B |
| 16. | Korber K. Quality assessment of economic evaluations of health promotion programs for children and adolescents-a systematic review using the example of physical activity. Health Econ Rev. 2015;5(1):35. | B |
| 17. | Gutiérrez-Ibarluzea I, Arana-Arri E. Nutrition, a health technology that deserves increasing interest among HTA doers. A systematic review. Front Pharmacol. 2015;6:156. | A |
| 18. | de Graaff B, Neil A, Sanderson K, Si L, Yee KC, Palmer AJ. A Systematic Review and Narrative Synthesis of Health Economic Studies Conducted for Hereditary Haemochromatosis. Appl Health Econ Health Policy. 2015;13(5):469-483. | F |
| 19. | Koleva-Kolarova RG, Zhan Z, Greuter MJ, Feenstra TL, De Bock GH. Simulation models in population breast cancer screening: A systematic review. Breast. 2015;24(4):354-363. | D |
| 20. | Oberjé EJ, de Kinderen RJ, Evers SM, van Woerkum CM, de Bruin M. Cost effectiveness of medication adherence-enhancing interventions: a systematic review of trial-based economic evaluations. Pharmacoeconomics. 2013;31(12):1155-1168. | E |
| 21. | Fenwick E, Macdonald C, Thomson H. Economic analysis of the health impacts of housing improvement studies: a systematic review. J Epidemiol Community Health. 2013;67(10):835-845. | A |
| 22. | Howard K. The cost-effectiveness of screening for anal cancer in men who have sex with men: a systematic review. Sex Health. 2012;9(6):610-619. | B |
| 23. | Ruger JP, Lazar CM. Economic evaluation of pharmaco- and behavioral therapies for smoking cessation: a critical and systematic review of empirical research. Annu Rev Public Health. 2012;33:279-305. | B |
| 24. | Wolfenstetter SB, Wenig CM. Economic evaluation and transferability of physical activity programmes in primary prevention: a systematic review. Int J Environ Res Public Health. 2010;7(4):1622-1648. | D |
| 25. | Tompa E, Dolinschi R, de Oliveira C, Irvin E. A systematic review of occupational health and safety interventions with economic analyses. J Occup Environ Med. 2009;51(9):1004-1023. | A |
| 26. | Müller-Riemenschneider F, Reinhold T, Willich SN. Cost-effectiveness of interventions promoting physical activity. Br J Sports Med. 2009;43(1):70-76. | B |
| 27. | Gebreslassie M, Sampaio F, Nystrand C, Ssegonja R, Feldman I. Economic evaluations of public health interventions for physical activity and healthy diet: A systematic review. Prev Med. 2020;136:106100. | B |
| 28. | Corrieri S, Heider D, Riedel-Heller SG, Matschinger H, König HH. Cost-effectiveness of fall prevention programs based on home visits for seniors aged over 65 years: a systematic review. Int Psychogeriatr. 2011;23(5):711-723. | D |
| 29. | Lynch M, Spencer LH, Tudor ER. A Systematic Review Exploring the Economic Valuation of Accessing and Using Green and Blue Spaces to Improve Public Health. Int J Environ Res Public Health. 2020;17(11):4142. | B |
| 30. | Mason AR, Hill RC, Myers LA, Street AD. Establishing the economics of engaging communities in health promotion: what is desirable, what is feasible?. Critical Public Health. 2008;18:3, 285-297. | C |
| 31. | Zechmeister I, Kilian R, McDaid D. MHEEN group. Is it worth investing in mental health promotion and prevention of mental illness? A systematic review of the evidence from economic evaluations. BMC Public Health. 2008;8:20. | B |
| 32. | Malottki K, Wang D, Andronis L, et al. Providing public health information to prevent skin cancer. West Midlands Health Technology Assessment Collaboration. 2009. | B |
| 33. | NICE. Supporting investment in public health: Review of methods for assessing cost-effectiveness, cost impact and return on investment. Proof of concept report: National Institute for Health and Care Excellence. 2011. | D |
| 34. | Jones L, Bates G, Downing J, Sumnall H, Bellis MA. A review of the effectiveness and cost-effectiveness of personal, social and health education in secondary schools focusing on sex and relationships and alcohol education for young people aged 11 to 19 years. United Kingdom: Centre for Public Health, Liverpool John Moores University. 2009. | B |
| 35. | Jones L, Bates G, Downing J, Sumnall H, Bellis MA. A review of the effectiveness and cost-effectiveness of personal, social and health education in primary schools focusing on sex and relationships and alcohol education for young people aged 5 to 11 years. Liverpool, Centre for Public Health, Liverpool John Moores University. 2009. | B |
| 36. | Jones L, Bates G, Downing J, Sumnall H, Bellis MA. A review of the effectiveness and cost effectiveness of alcohol and sex and relationship education for all children and young people aged 5–19 years in community settings. United Kingdom: Centre for Public Health, Liverpool John Moores University. 2010. | B |
| 37. | Jones L, James M, Jefferso T, et al. A review of the effectiveness and cost-effectiveness of interventions delivered in primary and secondary schools to prevent and/or reduce alcohol use by young people under 18 years old. Alcohol and schools: Review of effectiveness and cost-effectivenes. Main report. United Kingdom: Centre for Public Health, Liverpool John Moores University. 2007. | B |
| 38. | Lafortune L, Kelly S, Kuhn I, Cowan A, Brayne C. Disability, dementia and frailty in later life: mid-life approaches to prevent or delay the onset of these conditions. Report to NICE Public Health Guideline Committee. NICE. 2014. | B |
| 39. | Pelletier K R. Clinical and cost outcomes of multifactorial, cardiovascular risk management interventions in worksites: a comprehensive review and analysis. Journal of Occupational and Environmental Medicine 1997; 39(12): 1154-1169. | B |
| 40. | Pelletier K R. A review and analysis of the clinical- and cost-effectiveness studies of comprehensive health promotion and disease management programs at the worksite: 1998-2000 update. American Journal of Health Promotion 2001, 16(2), 107–116. | B |
| 41. | Pelletier KR. A review and analysis of the clinical and cost-effectiveness studies of comprehensive health promotion and disease management programs at the worksite: update VI 2000-2004. J Occup Environ Med. 2005;47(10):1051-1058. | B |
| 42. | Drummond Michael, Weatherly Helen, Ferguson Brian. Economic evaluation of health interventions BMJ 2008;337:a1204. | A |
| 43. | Unsal N, Weaver G, Bray J, Bibeau D.Public Health Rep. 2021 Feb 4:33354920976557. doi: 10.1177/0033354920976557. Online ahead of print.PMID: 33541206 | B |
| 44. | Nystrand C, Gebreslassie M, Ssegonja R, Feldman I, Sampaio F. A systematic review of economic evaluations of public health interventions targeting alcohol, tobacco, illicit drug use and problematic gambling: Using a case study to assess transferability. Health Policy. 2021 Jan;125(1):54-74. | B |
| 45. | Mahalingam M, Peterson C, Bergen G. Systematic review of unintentional injury prevention economic evaluations 2010-2019 and comparison to 1998-2009. Accid Anal Prev. 2020 Oct;146:105688. | B |
| 46. | Caldwell DM, Davies SR, Thorn JC, Palmer JC, Caro P, Hetrick SE, et al. School-based interventions to prevent anxiety, depression and conduct disorder in children and young people: a systematic review and network meta-analysis. Southampton (UK): NIHR Journals Library; 2021 Jul. | B |
| 47. | Le LK, Esturas AC, Mihalopoulos C, Chiotelis O, Bucholc J, Chatterton ML, Engel L. Cost-effectiveness evidence of mental health prevention and promotion interventions: A systematic review of economic evaluations. PLoS Med. 2021 May 11;18(5):e1003606. | B |
| 48. | Peterson C, Kearns MC. Systematic Review of Violence Prevention Economic Evaluations, 2000-2019. Am J Prev Med. 2021 Apr;60(4):552-562. | B |

A = no systematic or scoping review of full health economic studies

B = no methodological focus

C = no extractable data

D = focus on a specific study design (e.g. return on investment studies) or study type (e.g. CUA)

E = no focus on prevention/public health

F = specific nature of an intervention/disease
