# Supplementary material for: Methodological issues in economic evaluations of disease prevention and health promotion: an overview of systematic and scoping reviews
Source: BMC Public Health. 2021 Nov 20;21:2130. doi: 10.1186/s12889-021-12174-w (PMC8605499; doi:10.1186/s12889-021-12174-w)
Supplement: Supplementary file 5 — Additional file 5. Assessment of methodological aspects of the reviews included. [file 12889_2021_12174_MOESM5_ESM.docx]

# Additional File 5:

Assessment of methodological aspects of reviews included

| **Review** | **Objective**  **(1)** | **Inclusion criteria**  **(2)** | **Duplicate study selection and data extraction (3)** | **Literature search**  **(4)** | **Assessing the study quality**  **(5)** | **Discussion of heterogeneity**  **(6)** |
| --- | --- | --- | --- | --- | --- | --- |
| Cochrane et al. (2019) | To provide an overview of the methods used in economic evaluations of physical activity and sedentary behaviour interventions | - Full economic evaluations of interventions targeting individuals aged 16 years or over, who are defined as being physically inactive or sedentary - Interventions with minimum two observation time points - Trial and model based economic evaluations - Studies in English | - Study selection process: by two researchers - Duplicate data extraction based on a-priori defined criteria - Disagreements were resolved by discussion between two reviewers | - Databases: Medline via Ovid; SPORTSDiscus, EconLit and PsycINFO via EBSCOHost; National Health Service Economic Evaluations Database (NHS EED) and HTA via the Cochrane Library - Supplementary strategy used (e.g. key websites) - Key words provided - Publication years range: Jan 2009 -Mar 2017 | - Study quality: Drummond 10-point checklist - Results provided on the study level - Considering study quality by formulating conclusions: n.a. | - n.a. |
| Reeves et al. (2019) | To assess the extent to which economic evaluations have been applied to implementation interventions in public health and to assess their quality | - Full economic evaluations of implementation interventions applied to a public or population health area in a community setting - Interventions: any initiative designed to affect the uptake of DPHP-interventions in community settings - Studies in English | - Study selection process: by two researchers - Duplicate data extraction based on a-priori defined criteria - Disagreements between reviewers relating to study inclusion choices were resolved by consensus among all the reviewers | - Databases: MEDLINE, Embase, PsycINFO, CINAHL, Econ-Lit, EPPI-Centre database of health promotion research, Cost-Effectiveness Analysis Registry (CEA), NHS EED, Informit and Scopus - Supplementary strategy: hand search in the included studies - Key words provided - Publication years range: 1990-2017 | - Methodological quality: Drummond 10-point checklist - Reporting quality: Consolidated Health Economic Evaluation Reporting Standards (CHEERS) checklist - Methodological quality is considered in formulating conclusions | A quantitative synthesis including an analysis of heterogeneity was planned but not conducted due to different methodological aspects (e.g. time horizons or measures of effects) |
| Zanganeh et al. (2019) | To appraise the methods used in the economic evaluations of obesity prevention  and/or treatment interventions, to assess the quality of the economic evaluations, and to synthesise the evidence on the cost-effectiveness | - Full economic evaluations - Children and adolescents aged 0–19 years at the start of the intervention and/or their parents/guardians - All behavioural, environmental or policy interventions - No restrictions on the type of comparator or the outcome measure | - Study selection process: by two researchers - Data extraction process was checked for completeness and accuracy by an independent researcher - Consensus procedure for disagreements: resolved by discussion between all authors | - Eleven electronic databases (e.g. EMBASE) - Supplementary strategies used (e.g. Grey literature search) - Key words and MeSH terms provided - Publication years range: Jan 2001 -Apr 2017 | - Reporting quality: Drummond checklist - Reporting quality is considered in formulating conclusions | Heterogeneity in the methods (e.g. outcome measures ) used challenged the narrative synthesis and may therefore hinder comparability |
| Huter et al. (2018)^a^ | To examine to what extent requirements or methodological challenges of economic evaluations of health promotion or preventive interventions for older people are taken into account in applied analyses | - (i) target population ≥ 65 years (ii) interventions classified as HP/PP and (iii) full economic evaluation conducted. - Studies in English, Polish or German | - Study selection process: by two researchers - Duplicate data extraction - Consensus procedure for disagreements: n.a. | - Five electronic databases (e.g. EMBASE) - Supplementary strategies used (internet websites of 23 institutions/ projects related to the topic and reference lists of relevant papers) - Key words and MeSH terms provided - Publication years range: 2015-2018 | - Study quality was assessed with the Drummond checklist - Additionally criteria related to the special character of the economic evaluation of health promotion programs: i) societal perspective, ii) consideration of informal caregiving, productivity costs/unpaid work, iii) costs related to life years gained, iv) ‘beyond-health’ effects). - Results provided on the study level - Study quality is considered in formulating conclusions | - Economic evaluations performed are very heterogeneous in relation to cost categories included and the presentation of outcomes |
| Oosterhoff et al. (2018) | To examine the key aspects in the design of economic evaluations on school-based interventions targeting weight-related behaviours among 4–12 year olds, to discuss the main issues, and propose ways forward | - School-based interventions - Participants: children between 4-12 years (interventions eligible even if younger or older children were targeted simultaneously) - Full economic evaluation, societal cost-benefit analyses and societal return on investment analyses - Interventions targeting lifestyle behaviours (e.g. physical activity) | - Study selection process: by two researchers - Data extraction was conducted by one reviewer and independently checked by a second based on a-priori identified criteria - Disagreements about study inclusion were resolved by discussion between the reviewers | - Databases Medline via Pubmed and NHS EED - Supplementary strategies used (e.g. websites of relevant organizations or hand searches in the reference lists of included studies) - Key words and MeSH terms provided - Publication years range: from inception to Mar 2017 | - Reporting quality aligned with Dutch health economic guideline and the guidelines for social cost-benefit analysis - Results provided on the study level - Study quality is considered in formulating conclusions | -Variation in the scope of the studies (e.g. costs and outcomes beyond health) and the measurement, valuation, and extrapolation of costs and outcomes (e.g. little standardization in monetizing outcomes) mentioned |
| Dubas-Jakóbczyk-Jakobczyk et al. (2017 | To provide an overview on cost-effectiveness methods of health promotion and primary prevention interventions for older people | - (i) target population ≥ 65 years (ii) interventions classified as HP/PP and (iii) full economic evaluation conducted. - Studies in English, Polish or German | - Assessment upon inclusion criteria and data extraction by two researchers - Duplicate data extraction - Disagreements were resolved by seeking the opinion of the third researcher | - Five electronic databases (e.g. EMBASE) - Supplementary strategies used (internet websites of 23 institutions/ projects related to the topic and reference lists of relevant papers) - Key words and MeSH terms provided - Publication years range: 2000-2015 | - Study quality was assessed with the Drummond checklist - Results provided on the study level - Study quality is considered in formulating conclusions | Due to differences in the methods applied and the overall quality of the study, no deeper comparative analyses were performed |
| Hill et al. (2017) | To identify the methods of evaluation being used to appraise interventions to prevent excessive alcohol consumption and establish whether published studies provide sufficient information to meet the requirements of public health decision-makers. | - Full economic evaluations or methods of priority-setting (e.g. multi-criteria decision analysis) - Interventions preventing alcohol misuse or reducing excessive alcohol consumption - Studies in English | - Study selection and data extraction process: conducted by one and co-screened and verified, respectively, by other two researchers - Consensus procedure for disagreements: n.a. | - NHS Economic Evaluation Database (NHS EED) and Scopus - Supplementary strategies used (e.g. grey literature such as conference abstracts) - Key words and MeSH terms provided - Publication years range: Jan 2006 - May 2016 | - n.a. | n.a. |
| Döring et al.  (2016) | To explore existing methods and applications of economic evaluations, examining their limitations and making recommendations for future evaluations | - Trial-based or simulation-based cost-effectiveness analyses - Preschool children (<6 years) and/or their parents - Behavioural intervention targeting diet and physical activity - Outcome measures include at least one measure: body mass index, waist circumference or overweight prevalence - Studies available in English - Studies from European countries, USA, Canada, Australia, New Zealand | - Study selection process: by two researchers - Data extraction: was done by one author and checked by two other authors - Disagreements were resolved by discussion or the final judgement of a third reviewer | - Databases: Medline via PubMed, Cochrane Library, NHS EED and EconLit - Supplementary strategy: n.a. - Key words and MeSH terms provided - Publication years range: Jan 2004 to Nov 2015 | - Quality was assessed with a checklist developed by the Centre for Reviews and Dissemination of the University of York (adapted Drummond checklist) - A critical descriptive overview of key elements - Results provided on the study level - Consideration of quality assessment in formulating conclusions: n.a. | Due to heterogeneity in both application and methodology, a comparison of the economic findings and their translation into practice seems difficult and misleading |
| Alayli-Goebbels et al. (2014) | To review methodological quality of economic evaluations of lifestyle behavior change interventions and to examine how they addressed challenges in economic evaluation in this field | - Six key areas for behavior change: smoking, physical activity, dietary behavior, (illegal) drug use, alcohol use and sexual behavior - Studies available in English | - Study selection process: n.a. - Included studies were divided between six reviewers - Duplicate data extraction: n.a. - Consensus about questions arising during the data extraction and analysis process, were reached in regular meetings | - Databases: PubMed and the NHS EED - Supplementary strategy: n.a. - Key words and MeSH terms provided - Publication years range: until 2009 | - Methodological quality was assessed with the Drummond checklist - Results provided on the study level: n.a. - Methodological quality is considered in formulating conclusions | n.a. |
| Polinder et al. (2012) | To review and assess the quality of economic evaluation studies on injury prevention measures | - CEA, CBA and CUA studies of primary or secondary prevention of unintentional injuries - Studies available in English in a peer reviewed journal that could readily be accessed | - Study selection process: by two researchers - Duplicate data extraction - Disagreement about eligibility between the researchers was solved through discussion | - Databases: Medline (Pubmed), EMBASE, Web of Science, PsycINFO, and Safetylit - Supplementary strategy: reference lists of included articles - Key words provided - Publication years range: 1998-2009 | - Quality was assessed by the checklist of the Panel on cost-effectiveness in health and medicine (Gold et al., 1996) and by guidelines developed for economic submissions to the British Medical Journal - Results provided on the study level - Study quality is considered in formulating conclusions | Current differences in the design, reporting, and description of economic evaluations and in model assumptions, data definition and estimation, discount rates and perspectives, limit the ability to make head-to-head comparisons of the most effective and efficient injury prevention programs |
| Weatherly et al. (2009) | To identify methodological challenges of economic evaluations covering different public health areas (e.g. alcohol and drug use, obesity, smoking) | - (i) eleven public health areas, (ii) full economic evaluations, (iii) screening and immunization studies are excluded - Studies available in English | - Study selection process: by two researchers - Duplicate data extraction: n.a. - Consensus procedure for disagreements: n.a. | - Databases: NHS EED (incl. MEDLINE, EMBASE) - Supplementary search strategy used (e.g. hand search in working papers) - Key words provided - Publication years range: 2000-2005 | n.a. | n.a. |

CCA = cost-consequence analysis, CEA = cost-effectiveness analysis, CUA =cost-utility analysis, CMA =cost-minimization analysis , CBA = cost-benefit analysis, EA = , n.a. = not addressed, MeSH = Medical Subject Headings

^a^The review is based on the search strategy by Dubas-Jakóbczyk et al. 2017 (Eight of 37 studies are included in this overview).
